# Phenolic profile, *in vitro* antimicrobial and *in vivo* diuretic effects of endemic wild thyme *Thymus comosus* Heuff ex. Griseb. (Lamiaceae) from Romania

**DOI:** 10.3389/fphar.2023.1115117

**Published:** 2023-02-17

**Authors:** Mihai Babotă, Oleg Frumuzachi, Alexandru Nicolescu, Dejan Stojković, Marina Soković, Gabriele Rocchetti, Leilei Zhang, Luigi Lucini, Gianina Crișan, Andrei Mocan, Oliviu Voștinaru

**Affiliations:** ^1^ Department of Pharmaceutical Botany, “Iuliu Hațieganu” University of Medicine and Pharmacy, Cluj-Napoca, Romania; ^2^ Laboratory of Chromatography, Institute of Advanced Horticulture Research of Transylvania, University of Agricultural Sciences and Veterinary Medicine, Cluj-Napoca, Romania; ^3^ Department of Plant Physiology, Institute for Biological Research “Siniša Stanković”—National Institute of Republic of Serbia, University of Belgrade, Belgrade, Serbia; ^4^ Department of Animal Science, Food and Nutrition, Università Cattolica del Sacro Cuore, Piacenza, Italy; ^5^ Department for Sustainable Food Process, Università Cattolica del Sacro Cuore, Piacenza, Italy; ^6^ Department of Pharmacology, Physiology and Physiopathology, “Iuliu Haţieganu” University of Medicine and Pharmacy, Cluj-Napoca, Romania

**Keywords:** *Thymus comosus* Heuff. ex. Griseb., wild thyme, polyphenols, diuretic, antimicrobials

## Abstract

*Thymus comosus* Heuff ex. Griseb. (Lamiaceae) is a wild thyme species endemic for Romanian Carpathian areas, frequently collected as substitute for collective herbal product *Serpylli herba*, cited as antibacterial and diuretic remedy in traditional medicine. The present study aimed to evaluate the *in vivo* diuretic effect and *in vitro* antimicrobial properties of three herbal preparations (infusion—TCI, tincture—TCT and an hydroethanolic extract prepared through an optimized ultrasound-assisted method—OpTC) obtained from the aerial parts of *T. comosus* Heuff ex. Griseb, also evaluating their comprehensive phenolic profile. *In vivo* diuretic effect was tested using Wistar rats treated orally with each herbal preparation (125 and 250 mg/kg dispersed in 25 ml/kg isotonic saline solution) and quantified based on cumulative urine output (ml), diuretic action and diuretic activity. Additionally, sodium and potassium excretion were monitored using a potentiometric method with selective electrodes. *In vitro* antibacterial and antifungal activities were assessed using *p*-iodonitrotetrazolium chloride assay against six bacterial strains and six fungal strains by monitoring minimum inhibitory concentration (MICs), minimum bactericidal concentrations (MBCs) and minimum fungicidal concentrations (MFCs). Finally, phenolic profile of the aforementioned herbal extracts was evaluated using an ultra-high-pressure liquid chromatography (UHPLC) coupled with high-resolution mass spectrometry (HRMS) method to check the impact of the different preparations on the most abundant and significant compounds. All the extracts exerted a mild diuretic action, TCT and OpTC inducing the most intense diuretic effect. Both herbal preparations produced a statistically significant, dose-dependent and gradual increase of the urine output, the effect being more intense at 24 h (6.63–7.13 ml/24 h). Potentiometric evaluation of urine samples collected from treated rats revealed a clear and mild natriuretic and kaliuretic effect after the administration. In terms of antimicrobial activity, *E. coli* (MIC—0.38 mg/ml), *B. cereus* (MIC—0.75 mg/ml)), *Penicillium funiculosum* and *P. verrucosum* var. *cyclopium* (MIC—0.19 mg/ml) showed the greater sensitivity to the tested extracts, respectively. UHPLC-HRMS screening showed that the bioactive potential of *T. comosus* herbal preparations was likely related to the higher amounts of phenolic acids (including rosmarinic acid), flavonoids (mainly flavones and derivatives) and other phenolics (such as different isomers of salvianolic acids) in their composition. The obtained results support the ethnopharmacological evidence regarding the mild diuretic and antibacterial potentials of the endemic wild thyme *T. comosus*, this study being the first one that assessed the aforementioned bioactivities for this species.

## 1 Introduction

The benefits of herbal mild diuretic agents associated to the antimicrobial therapy for urinary tract infections (UTI) are supported by a great number of studies (Wright et al., 2007; Chandra et al., 2020; Das, 2020). UTI are the most common microbial disorders affecting both upper and lower segments of genito-urinary system, their prevalence being higher among females, elders and children; the main agents involved in the development of this condition are both bacterial (*Escherichia coli*, *Klebsiella* spp., *Proteus* spp., *Pseudomonas* spp.) and fungal (*Candida* ssp., *Cryptococcus* ssp.) strains, which show a variable sensitivity to the classic therapeutic agents (Chandra et al., 2020; Poloni and Rotta, 2020). Hence, in order to assure the success of therapy and decrease the overuse of antibiotics or antifungals (which leads to bacterial resistance) and their side effects, herbal remedies with antibacterial and diuretic activities are intensively promoted as potential alternatives for the treatment of UTI (Wright et al., 2007; Dobjanschi et al., 2018; Titko et al., 2020).

Diuresis is one of the main physiological processes involved in fluids homeostasis, its modulation being also linked with the management of several human pathologies, including hypertension, heart failure, urinary tract or hepatic disorders and poisonings (Zeng et al., 2019; Suri and Pamboukian, 2021; Zaccherini et al., 2021; Curtain et al., 2022; Kehrenberg and Bachmann, 2022; Zhou et al., 2022). Pharmacological evidence support the use of diuretics as valuable therapeutic agents in modern medicine, their pharmacodynamic and pharmacotoxicologic mechanisms being well-documented and, at the same time, carefully analyzed before the initiation of therapy (Titko et al., 2020; Kehrenberg and Bachmann, 2022). However, long-term use of some diuretics, especially in chronic diseases, is associated with several side-effects (i.e., hydroelectrolytic or hormonal imbalances, kidney injuries and hyperglycaemia) (Titko et al., 2020; Kehrenberg and Bachmann, 2022; Zhou et al., 2022), encouraging the development and use of alternative diuretic agents from natural sources, including phytomedicines.

In fact, research in the field of diuretics has roots in empirical observations of this effect after the administration of phytopreparations obtained from different natural sources. Dioscorides cited almost 105 herbs with diuretic potential in his famous work *De materia medica* (Yarnell and Touwaide, 2019), while traditional Chinese medicine or ayurvedic medicine mention the use of mixes containing various natural agents (including plants, fungi or minerals) able to increase the diuresis (Wright et al., 2007; Kasote et al., 2017). Moreover, based on ethnopharmacological evidence, these products could be officially recognized as effective therapeutic agents by the competent authorities. Committee on Herbal Medicinal Products (HMPC) of European Medicines Agency issued a monograph for the collective product *Species diureticae*, defined as a mix between maximum 4 herbal remedies from a list of 13 species with well-established or traditional use as diuretic agents (European Medicines Agency HMPC (a), 2017). The collective product and its individual herbal components are recommended as supportive treatment in a variety of conditions like inflammatory diseases of the lower urinary tract or renal gravel, in order to achieve flushing of urinary tract (European Medicines Agency HMPC (b), 2017).

*Thymus* species, including the spontaneous ones (currently recognized as “wild thyme”), are belonging to the most popular herbal antimicrobial agents, widely used in the development of phytomedicines indicated to alleviate cutaneous, respiratory or urinary infections. Beyond these uses, the additionally health-related benefits of wild thyme are supported especially by the ethnopharmacology, more other bioactivities for this product, including diuretic effects, being mentioned (Alarcón de la Lastra et al., 1987; Jarić et al., 2015; Li et al., 2019; Lorenzo et al., 2019; Patil et al., 2021). Aerial parts of wild thyme constitute the officinal product *Serpyllii herba* (*Serpylli herba* (Ph.Eur 10.8), 2022); according to pharmacopeial recommendation, the herbal drug could be collected only from *T. serpyllum* L. but, in current practice, substitution with other species is frequent due to low availability of the officinal one in different areas and its similar botanic features with other related *Thymus* species. Folk medicine of the Romanian Carpathian region describes the use of various wild thyme species, including *T. comosus* Heuff ex. Griseb., an endemic taxon of this area (Mărculescu et al., 2007; Pavel et al., 2010). Several studies previously evaluated potential bioactivities for *T. comosus*, including the antioxidant (Căpățină et al., 2021; Babotă et al., 2022), antibacterial (Pavel et al., 2009) or enzyme-inhibitory capacity (Babotă et al., 2022) of volatile and/or non-volatile phytocomplexes isolated from this species; however, at the moment, the available data still offer a scarce evidence on therapeutic benefits of *T. comosus* proven through *in vitro* and/or *in vivo* evaluations. At the same time, due to its limited use in the Intracarpathian areas, the only available ethnopharmacological evidence about the benefits of this species in the treatment of UTI is mostly supported through orally-transmitted tradition, documented by several authors (Butura, 1979; Grigorescu et al., 1986; Ciulei et al., 1993; Wagner, 1999; Pîrvu, 2006).

Hence, the main purposes of this study were (1) evaluation of phytochemical constituents of three different extracts obtained from the aerial parts of *T. comosus* (such as polyphenolic compounds, focusing on different subclasses), (2) diuretic and antimicrobial effects assessment of these herbal preparations and (3) the correlation analysis between chemical profile and the bioactive properties of this wild thyme species. Also, this work aims to support through experimental investigations the presumed value of this herbal product, used as remedy for urinary tract complaints in folk medicine mainly based on ethnopharmacogical evidence.

## 2 Material and methods

### 2.1 Plant material and extracts preparation

Aerial parts of *T. comosus* were collected in August 2019, during their maximum flowering period, from Rimetea (Alba County, Romania), being further dried at room temperature, kept away from sunlight. The authenticity of fresh plant material was confirmed based on its macroscopic characters by Dr. Andrei Mocan from Department of Pharmaceutical Botany, “Iuliu Hațieganu” University of Medicine and Pharmacy, voucher specimens being kept in the herbarium of Department (voucher no. 122.28.2.1). Dried material was ground to a fine powder (Ø <1 mm, standard sieve according to Ph. Eur 10.6) in order to assure optimal particle size for extraction; in order to evaluate also the influence of extraction procedure, three different extraction methods were used (Babotă et al., 2021, 2022):

*Infusion*: 500 ml of boiling water were added over 50 g of plant powder previously weighed in a Berzelius beaker, the mix being homogenized by manual stirring and kept for 30 min at room temperature.

*Tincture*: 500 ml of 70% *v/v* ethanol were added over 50 g of plant powder weighed in a ground joint Erlenmeyer flask, homogenized by manual stirring and kept for 10 days at room temperature in a dark place. In order to occur a proper maceration process, the mix was daily shaken for aprox. 1 min.

*Ultrasound-assisted extraction*: An optimized method previously developed was used (Babotă et al., 2022); 10 g of plant powder were mixed with 100 ml of 50% *v/v* ethanol, the extraction mixture being further ultrasonicated for 6.5 min (ultrasounds amplitude 34.8%) using an Vibra-Cell™ Ultrasonic Processor (model VCX 500). Five successive extractions were made in order to assure the same final amounts of the extracts like for infusion and tincture.

After extraction occurred, the supernatants resulted from each method were recovered through vacuum filtration, concentrated under reduced pressure and lyophilized, finally resulting three dried extracts which were further evaluated: TCI—*T. comosus* infusion, TCT—*T. comosus* tincture and OpTC—*T. comosus* optimized extract obtained by ultrasound-assisted extraction.

### 2.2 UHPLC-HRMS screening of phenolic compounds

The 3 different *T. comosus* dried extracts (100 mg each one) were dissolved in 2 ml of the corresponding extraction solvents, namely water (TCI), ethanol 70% (TCT), and ethanol 50% (OpTC). After centrifugation (6,000 × g, 10 min, at 4°C), the supernatants were transferred to HPLC vials and supposed to untargeted phenolic profiling through high-resolution mass spectrometry (HRMS) using a Q-Exactive™ Focus Hybrid Quadrupole-Orbitrap Mass Spectrometer (Thermo Scientific, Waltham, MA, United States) coupled to a Vanquish ultra-high-pressure liquid chromatography (UHPLC) pump, equipped with heated electrospray ionization (HESI)-II probe (Thermo Scientific, United States) (Babotă et al., 2022; Nicolescu et al., 2022). The post-acquisition data filtering was accomplished using MS-DIAL software (version 4.70), while the annotation was achieved via spectral matching against FoodDB and Phenol-Explorer databases. Overall, the mass accuracy (setting a 5-ppm tolerance for *m/z* values), isotopic pattern, and spectral matching were used to calculate a total identification score, considering the most common HESI + adducts for the chromatographic conditions adopted, thus reaching a level 2 of confidence in annotation.

Semi-quantitative appreciation of each previously annotated phenolic class was made by analyzing representative pure standard compounds under the same conditions: ferulic acid (phenolic acids), quercetin (flavonols), catechin (flavanols), cyanidin (anthocyanins), luteolin (flavones and other flavonoids), resveratrol (stilbenes), and oleuropein (other remaining phenolics). A linear fitting (*R* ^2^ > 0.99) was built and used for quantification, results being expressed as mg equivalents (Eq.)/g lyophilized extract (*n* = 3).

### 2.3 *In vivo* diuretic activity

#### 2.3.1 Animals

Forty-eight male Wistar rats with a medium weight of 178 ± 11 g were purchased from the Practical Skills and Experimental Medicine Centre of the Iuliu Haţieganu University of Medicine and Pharmacy, Cluj-Napoca, Romania. The animals were maintained in standard conditions (22°C ± 2°C, a relative humidity of 45% ± 10%, 12:12-h light:dark cycle), with free access to standard pelleted food (Cantacuzino Institute, Bucharest, Romania) and filtered water before the experiment, except for the day when the test substances were administered. The experimental protocol was approved by the Ethics Committee of the University, being compliant with the EU Directive 86/609/EEC, which regulates the use of laboratory animals for scientific research.

#### 2.3.2 Acute diuretic effect

The evaluation of the acute diuretic effect of TCI, TCT and OpTC was performed using isotonic saline solution as hydrating fluid (Kau et al., 1984). Forty-eight Wistar rats randomized in eight groups (*n* = 6) were used. The rats from the control group were treated orally with 25 ml/kg isotonic saline solution (Braun, Germany), while the rats from the reference group were treated orally with 10 mg/kg furosemide (Zentiva, Romania), dissolved also in a volume of 25 ml/kg isotonic saline solution. Two groups of rats were treated orally with 125 and 250 mg/kg TCI dispersed in a volume of 25 ml/kg isotonic saline solution, while other two groups of rats received also orally 125 and 250 mg/kg TCT dispersed in 25 ml/kg isotonic saline solution. Finally, two groups of rats were treated orally with 125 and 250 mg/kg OpTC dispersed in the same volume of 25 ml/kg isotonic saline solution.

Afterwards, the animals were individually placed in metabolic cages, the environmental temperature being maintained at 22°C. The cumulative urine output (ml) was recorded for each animal at two different time intervals: 5 h and 24 h after the administration of a single dose from the tested substances (Zhang et al., 2010). Two key parameters of diuretic effect were calculated 24 h after the substance administration: diuretic action, as the ratio of urine output in the test groups to urine output in the control group and diuretic activity, as the ratio of urine output in the test groups to urine output of the reference group.

#### 2.3.3 Electrolyte excretion

In order to evaluate electrolyte excretion, urinary concentration of sodium and potassium ions (U_Na_ and U_K_) was determined in the collected urine samples, 5 h and 24 h after the substance administration, by a potentiometric method with selective electrodes, using a VITROS 250 Chemistry System automatic analyzer (Johnson and Johnson Clinical Diagnostic), being expressed in mEq/kg (Voștinaru et al., 2017; Babotă et al., 2021).

### 2.4 *In vitro* antimicrobial potential

The extracts were tested for their antibacterial potential against Gram-positive bacteria *Staphylococcus aureus* (ATCC 11632), *Bacillus cereus* (clinical isolate), *Listeria monocytogenes* (NCTC 7973), as well as the following Gram-negative bacteria: *Escherichia coli* (ATCC 25922), *Salmonella* Typhimurium (ATCC 13311) and *Enterobacter cloacae* (ATCC 35030). For the antifungal assays, six micromycetes were used, namely *Aspergillus fumigatus* (human isolate), *Aspergillus niger* (ATCC 6275), *Aspergillus versicolor* (ATCC11730), *Penicillium funiculosum* (ATCC 36839), *Penicillium verrucosum* var. *cyclopium* (food isolate) and *Trichoderma harzianum* (TH-IS005-12). All strains were obtained from the Mycological Laboratory, Department of Plant Physiology, Institute for Biological Research “Siniša Stanković”, University of Belgrade, Serbia. A modified microdilution technique was employed to examine the extracts’ antibacterial activity (CLSI. Clinical and Laboratory Standards Institute, 2009). In Luria broth medium, bacterial species were grown for 24 h at 37°C. With sterile 0.85% saline that contained 0.1% Tween 80 (v/v), the fungal spores were clollected from the surface of the agar plates that were previously inoculated with microfungi and grown for 21 days at 28°C. Sterile saline was used to adjust the bacterial cells and fungal spore suspension to a concentration of roughly 1.0 × 10^5^ CFU in a final volume of 100 μL per each well. For later use, the inocula were kept in storage at 4°C. To ensure there was no contamination and to confirm the validity of the inoculum, dilutions of the inocula were cultured on Mueller-Hinton agar for bacteria and solid malt agar for fungi.

A serial dilution procedure was used to determine the minimum inhibitory concentration (MIC) in 96-well microtiter plates. The studied extracts were dissolved in 30% EtOH (20 mg/ml) and added to broth malt medium (for fungi) or Luria broth medium (for bacteria) with the inocula. The microplates were incubated for 24 h at 37°C for bacteria and 72 h at 28°C for fungi. The following day, 30 μL of INT (p-iodonitrotetrazolium violet) solution 0.2 mg/ml was added to tested bacteria containing medium, and the plates were placed back in the incubator for at least 30 min to guarantee a sufficient color reaction (Tsukatani et al., 2012). A clear solution or a distinct decrease in color response were indicators of growth inhibition. MICs were established as the lowest concentrations at which there was no discernible growth (under the binocular microscope). By serially subcultivating a 2 μL sample into microtiter plates with 100 μL of broth in each well and then incubating the plates for 24 h or 72 h at 28°C or 37°C, the minimum bactericidal concentrations (MBCs) and minimum fungicidal concentrations (MFCs) were found. MBC/MFC, which standed for the lowest concentration with no discernible growth and represented a 99.5% killing of the original inoculum. Plant extracts with MICs <0.1 mg/ml were considered highly active antimicrobial agents, while MICs ˃ 1 mg/ml were considered as inactive (Kokoska et al., 2019). The experiments were repeated in triplicate for each concentration used. A 30% EtOH negative control was employed. Ampicillin and streptomycin (Panfarma, Belgrade, Serbia) were used as positive antibacterial controls (0.1–1 mg/ml). Commercial fungicides bifonazole and ketoconazole (0.1–1 mg/ml) (Srbolek, Belgrade, Serbia) were used as positive controls; all results were expressed as mg/mL (Añibarro-Ortega et al., 2021; García-Pérez et al., 2021).

### 2.5 Statistical analysis

The semi-quantitative data from phenolic profiling were subjected to analysis of variance (ANOVA) considering the impact of the different extraction type. Means and standard deviation were then calculated from triplicates (*n* = 3). The mean values were compared by Tukey’s test, with a significance level of 95% (*p* < 0.05) using the IBM PASW Statistics 27.0 (SPSS Inc.). Regarding multivariate data elaboration of metabolomics features, the analysis was done using two different software, namely MetaboAnalyst (Pang et al., 2022) and SIMCA 13 (Umetrics, Malmo, Sweden). Briefly, data were median-centered, Pareto scaled, and log2-transformed before building unsupervised and supervised models, namely hierarchical cluster analysis (HCA, Euclidean distance), principal component analysis (PCA), and orthogonal projections to latent structures discriminant analysis (OPLS-DA). The OPLS-DA model validation parameters (goodness-of-fit R^2^Y and goodness-of-prediction Q^2^Y) were also recorded. Each discriminant model was inspected for outliers, cross-validated (CV-ANOVA), and the permutation testing (N = 200) excluded over-fitting. The importance of each compound for discrimination was then evaluated using the variables importance in projection (VIP) selection method, using a VIP score threshold of >1 (meaning extremely discriminant in the orthogonal projection).

## 3 Results

### 3.1 Untargeted phenolic profiling of the extracts

In this work, the UHPLC-HRMS profiling allowed us to putatively annotate several phenolic compounds, including 48 anthocyanins, 68 flavones and other flavonoids, 25 flavan-3-ols, 51 flavonols, 39 other phenolics, 36 phenolic acids, and 5 stilbenes. The compounds annotated against the databases FooDB and Phenol-Explorer are reported in supplementary material considering their relative abundance values, isotopic MS, and MS/MS spectra. In our experimental conditions, *T. comosus* was found to be numerically abundant in flavonoids (recording a total of 192 compounds), following by the group consisting in other phenolics and phenolic acids (several hydroxycinnamic and hydroxybenzoic derivatives). Also, the QC (quality control) samples (analysed under MS/MS analysis) allowed us to structurally confirm the identity of 77 compounds (28% of total phenolics annotated), and among the most abundant compounds we found several flavonoids, such as luteolin-7-galactoside (a flavone), cirsilineol (a flavone), 3-methoxynobiletin (a flavonol), and sakuranetin (a flavanone) (supplementary material). Besides, typical *Thymus* metabolites were annotated and structurally confirmed, including isomers of salvianolic acid (such as salvianolic acids C and D), gallic acid (belonging to hydroxybenzoic acids) and the flavanone eriodictyol-7-glucoside. In the next step, the annotated phenolics were quantified according to pure standard compounds, considered as representative of the identified phenolic classes. The semi-quantitative analysis from UHPLC-HRMS data (Table 1) outlined significant differences regarding the quantitative distribution of phenolic compounds in the analyzed samples, strongly influenced by the solvents and the extraction methods used. Overall, infusion (TCI) showed the lowest values in terms of total phenolic content (1,433.86 μg/g), while for maceration (TCT) the amount of quantified phenolics was 1.31-fold higher. Besides, ultrasound-assisted extraction (OpTC) allowed the best recovery of phytoconstituents from *T. comosus* aerial parts, showing the highest concentration of total phenolic compounds (2075.82 μg/g). Looking at specific phenolic subclasses, OpTC recorded the highest values for anthocyanins, flavonols, other phenolics, and stilbenes (Table 1). However, the highest phenolic acids content was observed in TCI samples, followed by TCT and OpTC extracts. Finally, maceration allowed the best recovery of flavones (904.56 μg/g) while TCI promoted a better extraction of flavan-3-ols (50.06 μg/g).

**TABLE 1:** Semi-quantitative analysis on the total phenolic content in the different *T. comosus* extracts considering the main phenolic subclasses, namely anthocyanins, flavones and other flavonoids, flavonols, flavan-3-ols, phenolic acids, other phenolics, and stilbenes. Results are expressed as mean value (µg Equivalents/g dry matter) ± standard deviation (*n* = 3). TPC = total phenolic content; Eq. = Equivalents. Superscript letters within each row indicate significant differences (Duncan’s test; *p*-value <0.05).

Phenolic class	OpTC (µg/g)	TCI (µg/g)	TCT (µg/g)
Anthocyanins	94.35 ± 1.86^c^	68.15 ± 3.59^a^	81.07 ± 1.23^b^
Flavones and other flavonoids	794.13 ± 0.52^b^	492.57 ± 6.29^a^	904.56 ± 7.18^c^
Flavonols	206.17 ± 1.36^c^	83.23 ± 0.28^b^	63.32 ± 1.32^a^
Flavan-3-ols	44.58 ± 3.78^a^	50.06 ± 0.82^b^	40.41 ± 2.33^a^
Phenolic acids	97.03 ± 7.50^a^	154.61 ± 0.68^b^	120.09 ± 19.61^a^
Other phenolics	797.98 ± 55.13^c^	564.64 ± 31.73^a^	660.33 ± 15.28b
Stilbenes	41.57 ± 8.09^b^	20.60 ± 5.12^a^	15.81 ± 6.19^a^
TPC	2075.82	1,433.86	1885.59

**FIGURE 1:**
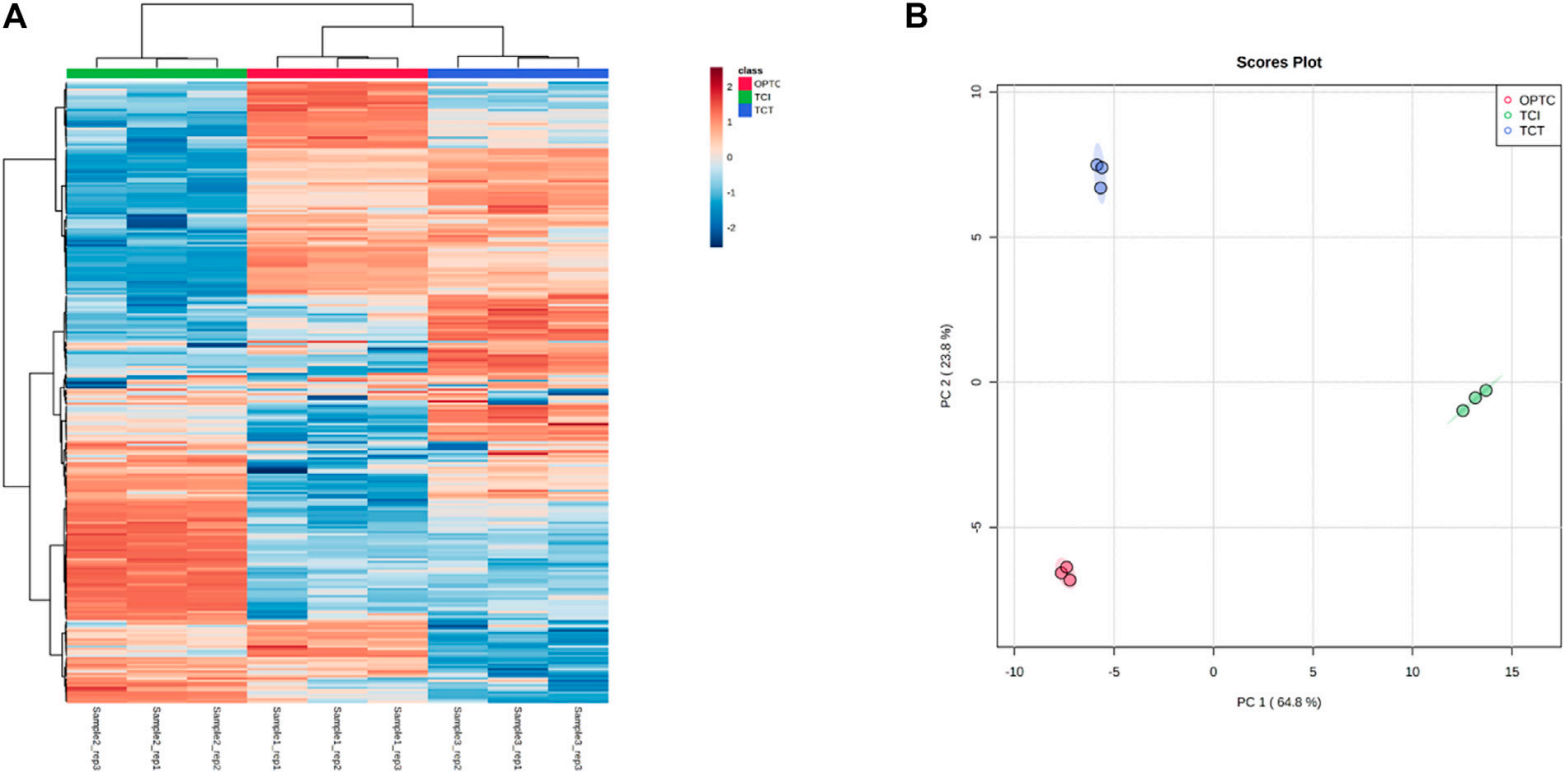
Unsupervised multivariate statistics built considering the phenolic profiles of OpTC, TCI, and TCT sample extracts. **(A)** = heat map based on not averaged hierarchical cluster analysis; **(B)** = principal component analysis score plot.

The differences between the three extraction methods were then inspected by using both unsupervised and supervised multivariate statistical approaches. As unsupervised tools, the hierarchical clustering (HCA) and principal component analysis (PCA) were selected, while the OPLS-DA approach was used as supervised tool to extrapolate the discriminant compounds of each extraction method (i.e., OpTC, TCI, and TCT). Figures 1A, B reports the outputs of both HCA heat map and PCA score plot, respectively. The HCA heat map, built considering the log2 fold-change variation of each phenolic compound across each sample replicate, allowed us to clearly discriminate the TCI vs*.* OpTC and TCT samples, and the sample information was obtained by inspecting the PCA score plot, with two principal components explaining the 88.6% of the total variability. It was interesting to notice that each extraction method tested provided exclusive up- and down-accumulated set of compounds, as revealed by the red and blue colors of the heat-map (Figure 1A).

**FIGURE 2:**
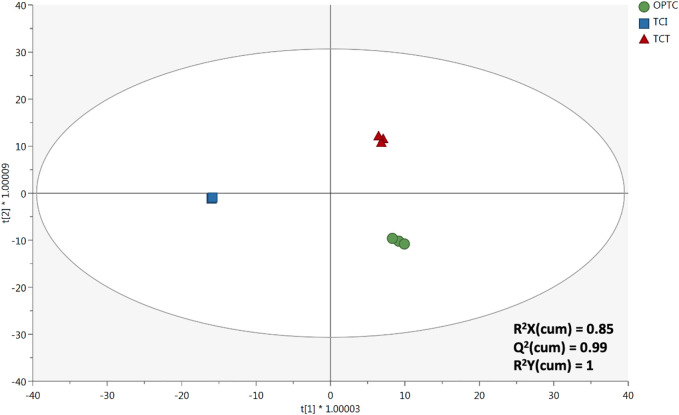
Supervised OPLS-DA built considering the untargeted phenolic profile of *T. comosus* following different extraction methods, namely OpTC, TCI, and TCT.

Therefore, the unsupervised analyses confirmed the ability of phenolic compounds to be potential markers of the extraction processes under investigation (i.e., OpTC, TCI, and TCT). Thereafter, to extrapolate the discriminant compounds of the trends observed (Figure 1), we used a supervised OPLS-DA approach followed by a VIP selection method of the most discriminant variables (compounds). As can be observed from Figure 2, the orthogonal latent vector clearly separated TCI from the other two extraction systems, thus allowing to confirm the unsupervised findings. The supervised model consisted in very accurate and robust goodness parameters, proven by the Q^2^ (prediction ability) value equal to 0.989. Besides, no significant outliers in the prediction model were observed (supplementary material). Interestingly, several phenolic compounds (176) showed a great discriminant potential, with 9 phenolics possessing a VIP score higher than 1.15, such as 6-hydroxykaempferol-3-glucoside (marker of OpTC), xanthotoxin (marker of OpTC), delphinidin 3-feruloylglucoside (marker of both TCT and TCI), paeoniflorin (marker of OpTC), conidendrin (marker of TCT and TCI), naringenin 5-glucoside (marker of OPTC), epigallocatechin 3-*p*-coumarate (marker of OpTC and TCI), luteolin 7-galactoside (OpTC and TCI), and malvidin 3-alpha-L-galactoside (marker of TCT). All the VIP marker compounds can be found in supplementary material file combined in a detailed list.

### 3.2 *In vivo* diuretic activity

**FIGURE 3:**
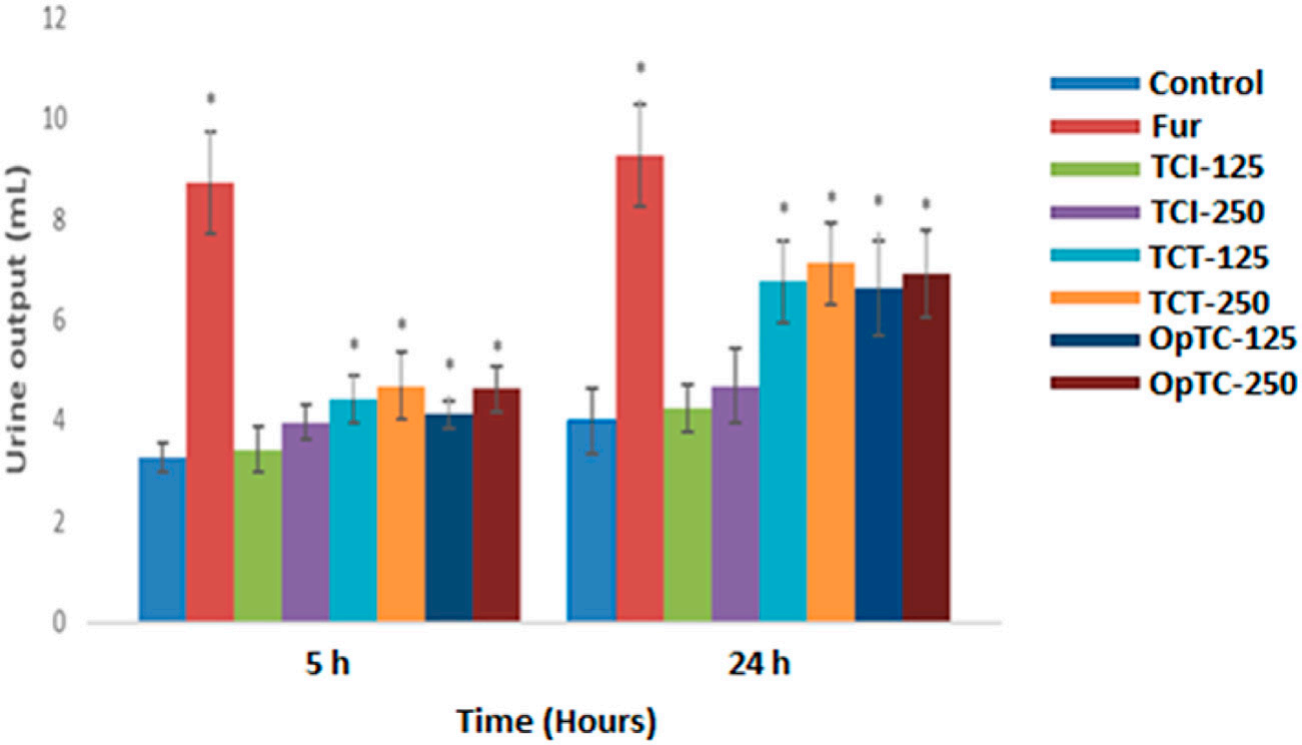
Effect of herbal preparations obtained from *Thymus comosus* on urine output recorded at 5 and 24 h in saline loaded Wistar rats (*p < 0.05 vs. saline control).

As shown in Figure 3, OpTC and TCT produced a statistically significant, dose-dependent and gradual increase of the urine output, the effect being more intense at 24 h. TCT (250 mg/kg) exerted the most intense diuretic effect with an urine output of 7.13 ± 0.81 ml at 24h, while at the same dose, TCI’s activity was clearly reduced.

Additionally to urine output, diuretic effect was quantified based on other two parameters, namely diuretic action and diuretic activity (described in Table 2). Diuretic action ranged from 1.69 to 1.78 for the animals treated with TCT and from 1.65 to 1.72 for the animals treated with OpTC. The strongest diuretic activity was observed in the animals treated with TCT at 250 mg/kg, which showed 76% of the activity of furosemide, the reference diuretic drug.

**TABLE 2:** Parameters of the diuretic effect in saline loaded Wistar rats treated with herbal preparations obtained from *Thymus comosus*. Values of urine output are expressed as Mean ± SD (*n* = 6); (*p < 0.05 vs. saline control).

Group (dose)	Urine output at 24 h (ml)	Diuretic action	Diuretic activity
Control (saline solution)	4 ± 0.64	—	—
Furosemide (10 mg/kg)	9.26 ± 1.02	2.31	1
TCI 125 mg/kg	4.25 ± 0.48	1.06	0.45
TCI 250 mg/kg	4.70 ± 0.74	1.17	0.50
TCT 125 mg/kg	6.76 ± 0.82*	1.69	0.73
TCT 250 mg/kg	7.13 ± 0.81*	1.78	0.76
OpTC 125 mg/kg	6.63 ± 0.95*	1.65	0.71
OpTC 250 mg/kg	6.91 ± 0.86*	1.72	0.74

The urinary excretion of Na^+^ and K^+^ ions (Table 3) produced by TCT and OpTC, was statistically significant, being superior at 24 h. The most significant excretion of the tested electrolytes was produced by TCT (250 mg/kg) with U_Na_ and U_K_ values of 3.88 ± 0.55 and 2.63 ± 0.39 mEq/kg, 24 h after the substance administration. The calculated Na^+^/K^+^ ratio for TCT and OpTC treated groups did not show values above 10 at any moment of determination, thus indicating a lack of a potassium-sparing effect, like for furosemide. In fact, both TCT and OpTC produced a clear but mild natriuretic and kaliuretic effect, while as well as for urine output, TCT had a lower effect on potassium excretion.

**TABLE 3:** Effect of the herbal preparations obtained from *Thymus comosus* on urinary excretion of sodium (U_Na_) and potassium (U_K_) 5 h and 24 h after the substance administration, and the ratio Na/K in saline loaded Crl:WI rats. Values of U_Na_V and U_K_V are expressed as Mean ± SD (*n* = 6); (*p < 0.05 vs. saline control).

Group (dose)	U_Na_ at 5 h (mEq/kg)	U_K_ at 5 h (mEq/kg)	U_Na_ at 24 h (mEq/kg)	U_K_ at 24 h (mEq/kg)	Na/K at 24 h
Control (saline)	1.20 ± 0.25	1.08 ± 0.11	1.50 ± 0.21	1.42 ± 0.13	1.05
Furosemide (10 mg/kg)	4.23 ± 0.65*	3.08 ± 0.24*	4.77 ± 0.68*	3.21 ± 0.39*	1.48
TCI (125 mg/kg)	1.33 ± 0.42	1.21 ± 0.17	1.67 ± 0.32	1.54 ± 0.23	1.08
TCI (250 mg/kg)	1.52 ± 0.43	1.39 ± 0.29	1.82 ± 0.31	1.63 ± 0.44	1.11
TCT (125 mg/kg)	2.08 ± 0.63*	1.75 ± 0.55*	2.97 ± 0.45*	2.04 ± 0.21*	1.45
TCT (250 mg/kg)	3.21 ± 0.46*	2.27 ± 0.39*	3.88 ± 0.55*	2.63 ± 0.39*	1.47
OpTC (125 mg/kg)	2.84 ± 0.51*	1.93 ± 0.12*	3.06 ± 0.53*	2.18 ± 0.42*	1.40
OpTC (250 mg/kg)	3.02 ± 0.68*	2.15 ± 0.13*	3.45 ± 0.51*	2.36 ± 0.29*	1.46

### 3.3 *In vitro* antimicrobial potential

As it could be observed in Table 4, our assessment proven overall a weak antibacterial activity for all herbal preparations obtained from *T. comosus* aerial parts. MIC and MBC values indicated TCI and TCT as being slightly active against *E. coli* (MICs of 0.38 mg/ml), while OpTC exerted a weak effect against *B. cereus* (MIC value of 0.75 mg/ml); conversely, a lack of activity was proven for *S. aureus*, *L. monocytogenes* and *E. cloacae*. Regarding the differences shown between the extracts in terms of antibacterial potency, OpTC showed lower MICs for Gram-positive strains (*S. aureus*, *B. cereus* and *L. monocytogenes*), while TCT and TCI exerted the most effective activity against *E. coli*.

**TABLE 4:** MIC and MBC values (mg/ml) of the herbal preparations obtained from *Thymus comosus* after the evaluation of their antibacterial potential.

		*S. aureus (ATCC 11632)*	*B. cereus (clinical isolate)*	*L. mono-cytogenes (NCTC 7973)*	*S.* Typhimurium *(ATCC 13311)*	*E. coli (ATCC 25922)*	*E. cloacae (ATCC 35030)*
TCI	MIC	6	1.5	6	1.5	0.38	3
	MBC	12	3	12	3	0.75	6
TCT	MIC	6	1.5	6	1.5	0.38	3
	MBC	12	3	12	3	0.75	6
OpTC	MIC	3	0.75	3	1.5	3	3
	MBC	6	1.5	6	3	6	6
Streptomycin	MIC	0.1	0.025	0.15	0.1	0.1	0.025
	MBC	0.2	0.05	0.3	0.2	0.2	0.05
Ampicillin	MIC	0.1	0.1	0.15	0.1	0.15	0.1
	MBC	0.15	0.15	0.3	0.2	0.2	0.15

The antifungal effect was also proven for *T. comosus* extracts, their MIC and MFC being summarized in Table 5. *Penicillium* species were found as the most sensible fungal strains, this trend being observed for all tested phytopreparations. The highest MIC and MFC values were measured for *A. versicolor*, which were correlated with a weak sensitivity of this strain. Interestingly, in comparison with the antibacterial effect, the extraction procedure seems to have a low influence on the antifungal properties of *T. comosus* extracts, all tested herbal preparations exerting similar MICs and MBCs after the evaluation of their ability to inhibit fungal growth of each individual strain.

**TABLE 5:** MIC and MFC values (mg/ml) of the herbal preparations obtained from *Thymus comosus* after the evaluation of their antifungal potential.

		*A. fumigatus* (human isolate)	*A. niger* (ATCC 6275)	*A. versicolor (ATCC11730)*	*P. funiculosum (ATCC 36839)*	*P. verrucosum* var. *cyclopium (food isolate)*	*T. harzianum (TH-IS005-12)*
TCI	MIC	0.75	6	0.75	0.19	0.19	0.75
	MFC	1.5	12	1.5	0.38	0.38	1.5
TCT	MIC	0.75	6	0.75	0.19	0.19	0.75
	MFC	1.5	12	1.5	0.38	0.38	1.5
OpTC	MIC	0.75	6	0.75	0.19	0.19	0.75
	MFC	1.5	12	1.5	0.38	0.38	1.5
Bifonazole	MIC	0.15	0.1	0.15	0.2	0.1	0.1
	MFC	0.2	0.2	0.2	0.25	0.2	0.2
Ketoconazole	MIC	0.2	0.2	0.2	0.25	0.2	1
	MFC	0.5	0.5	0.5	0.5	0.3	1.5

## 4 Discussion

In the context of actual trends regarding the development of new or alternative therapeutic agents among natural sources, ethnopharmacological evidence could be considered as valuable hypotheses which support the background of pre-clinical or clinical research in the field of plant-based medicines. Moreover, sometimes, the only available data about the potential applications of endemic species in phytotherapy are those supplied *via* ethnopharmacology, considering their limited use by indigenous populations in well-defined areas. *T. comosus* is mentioned as an endemic taxa for Carpathian areas of Romania, without being listed as endangered species (Sîrbu et al., 2013; Hurdu et al., 2022). In Romanian folk medicine, it is frequently assigned as “*cimbrișor*" or *“cimbru de câmp*" (Butura, 1979), both terms defining a group of wild thyme species which are the main source for herbal product *Serpylli herba*. Even though the product is recommended as antibacterial and mild diuretic agent based on its traditional use, there are very few studies which prove these bioactivities based on *in vitro* or/and *in vivo* evaluations; besides, after a literature survey, the only *in vivo* assessment available for the diuretic potential of *Thymus* species was realized on *T. carnosus* Boiss. (Alarcón de la Lastra et al., 1987).

Hence, the present study emphasizes for the first time the potential benefits associated to the use of *T. comosus* herbal preparations in the treatment of urinary tract infections due to their dual action, antimicrobial and mild diuretic one respectively. Of course, antimicrobial potential of other *Thymus* species was previously proven, being attributed both to the volatile oils found in their aerial parts (rich in thymol and carvacrol) and to the non-volatile fractions which contain high amounts of various types of phenolic compounds (Lorenzo et al., 2019; Salehi et al., 2019; Tohidi et al., 2019). As we already mentioned, the untargeted phenolic profiling (realised by using an UHPLC-HRMS approach) revealed important amounts of several phenolic classes, mainly flavonoids and phenolic acid derivatives for all the analyzed samples, including luteolin derivatives, followed by phenolic acids and other phenolics, including rosmarinic acid and salvianolic acids (such as C, D, and L). These findings are in strict agreement with previous published results on *T. comosus* phytochemical profile (Mărculescu et al., 2007; Căpățină et al., 2021; Babotă et al., 2022). Also, the presence of the above-mentioned compounds in *T. comosus* likely affected their antibacterial effect which, as it could be observed in Table 4, was correlated with the semi-quantitative distribution of several phenolic subclasses, such as flavones and other flavonoids, other phenolics (including salvianolic acids and tyrosol derivatives), and phenolic acids (such as rosmarinic acid and gallic acid). The multivariate statistical analyses revealed that each extraction method tested (i.e., OpTC, TCI, and TCT) allowed the best recovery of certain compounds. For example, rosmarinic acid was a marker of the TCT, recording a VIP score equal to 1.11. This compound is an hydroxycinnamic acid derivative commonly found in plants belonging to Lamiaceae family (including *Thymus* species), which was already confirmed as potent antibacterial agent (Nadeem et al., 2019; Zhang et al., 2022). The most recent evidences suggest complex mechanisms involved in the antibacterial activity of this compound, including inhibition of bacterial proteins synthesis, induction of bacterial cells walls rupture, as well as the inhibition of Na^+^/K^+^-ATP-ase from bacterial membranes; all these effects were previously proven for *E. coli*, *S. aureus*, *Salmonella* ssp. and *B. subtilis*, *E. coli* showing the highest sensitivity among all tested strains (Zhang et al., 2022). Moreover, rosmarinic acid is also cited as promising inhibitor of fungal growth, its ability to reduce fungal biofilm formation or to act as membrane or mitochondrial disruptor being recently highlighted (Ivanov et al., 2022). Antimicrobial properties of various *Thymus* species are well-documented in literature, the sensitivity of different bacterial and fungal strains being tested both for volatile oils, as well as for non-volatile fractions. Three hydroethanolic extracts (prepared through maceration, heat-assisted and ultrasound-assisted extraction) obtained from the aerial parts of *T. serpyllum* were tested against *E. coli* (ATCC 25922) using a similar method, all of them showing a MIC value of 5 mg/ml (Jovanović et al., 2021); the same strain was evaluated for hydroethanolic extracts of *Thymus marschallianus* and *Thymus seravschanicus*, with a MIC value of 10 mg/ml (Zhumakanova et al., 2021). Hence, even though our results are emphasizing a weak activity for *T. comosus* phytopreparations, this is still higher than that one previuosly reported for similar extracts obtained from other related thyme species. Unfortunately, no significant relationship between any of the extract’s components or classes of compounds and the extract’s antimicrobial effect was noted, indicating that the extracts antimicrobial activities were the result of the synergistic/antagonistic effects of all of their specific constituents and quantitative presence, which can be the subject of further in-depth investigations regarding the antimicrobial effects of *T. comosus*. Interestingly, we found that TCI allowed the best recovery of two salvianolic acids, namely salvianolic acid L (VIP score = 1.02) and salvianolic acid D (VIP score = 1.05), whilst TCT was the best extraction system to recover the highest amount of salvianolic acid C (VIP score = 1.10). Salvianolic acids are phenolic compounds characterized by a high water-solubility, therefore the results obtained in this work are in line with the chemical behavior of these compounds (Ho and Hong, 2011). However, the salvianolic acids family is widely studied because of different therapeutic activities, including radical scavengers during cardiovascular injury, inhibition of leukocyte-endothelial cell adherence, inhibition of inflammation during cardiovascular injury, and immunomodulation (Ho and Hong, 2011). Besides, salvianolic acids (such as A and B) are reported in scientific literature to possess not only anti-inflammatory and anticancer actions, but also antibacterial and antiviral activities (Fu et al., 2021).

On the other side, the assessment of diuretic potential for the extracts obtained from *T. comosus* is another main achievement of our work, the evaluated parameters supporting the traditional medicine recommendations of this species as potential treatment for UTI. As well as for the antimicrobial activity, it is difficult to appreciate if the diuretic effects of *T. comosus* extracts are occurring from the individual influence of some chemical constituents or there is also an synergistic effect derived from the phytocomplexes found in this herb Various herbal products have a well-established and documented use as remedies for infectious diseases associated to urinary tract, the most popular of them being bearberry (*Arctostaphylos uva-ursi* L.) and lingonberry (*Vaccinium vitis-idea* L.) leaves, cranberry (*Vaccinium macrocarpon*) fruits, *Solidago virgaurea* L. (goldenrod), and *Equisetum arvense* L. (horsetail) aerial parts (Chandra et al., 2020; Das, 2020). Additionally to the antimicrobial effects exerted by the phytochemicals found in these herbs, other mechanisms (i.e., increase of urine production or urine pH) could be associated to their use in order to decrease the pathogens proliferation and the clinical symptoms (Chandra et al., 2020). As we already mentioned, there is a lack of knowledge regarding the *in vivo* diuretic potential of *Thymus* species, but this bioactivity was proven for other plants belonging to Lamiaceae family with a similar phytochemical profile (Michel et al., 2020). Arafat et al. proven the diuretic activity of *Orthosiphon stamineus* Benth. leaves, an herbal product with an high content of rosmarinic acid, obtaining 24 h urine output values between 4.9 and 5 ml for methanolic and hydromethanolic extracts (Arafat et al., 2008). In a similar way, *Ajuga* sp. (i.e. *A. integrifolia* Buch., *A. remota* Benth.) leaves and aerial parts (rich in phenolic acids) were found as potent *in vivo* diuretics in rodent studies (Hailu and Engidawork, 2014; Chilo and Raju, 2021). Diuretic action measured in a rat study for *A. remota* fractions (in doses of 250, 500 and 1,000 mg/kg) revealed values between 1.05 and 2.53, while diuretic activity was shown as varying between 0.41 and 0.99 in comparison with positive control, furosemide respectively (Chilo and Raju, 2021). According to previous published works (Gujral et al., 1955; Hailu and Engidawork, 2014), a good diuretic activity is described by values above 1.5, but as long as there are no other reports regarding diuretic effects of other thyme species, we prefer to be cautious and define *T. comosus* as a mild diuretic agent, this statement being a good start point for further confirmations regarding this effect by human studies. Based on the same reports we can also suppose that same compounds (phenolic acids derivatives, including rosmarinic acid) could be responsible for the diuretic potential of *T. comosus*. Hence, it can be supposed that the mild diuretic effect of *T. comosus* could be exploited as synergic with the already-proven antibacterial one for the treatment of UTI. Moreover, the herbal drug seems to be a safe one from the perspective of side effects on hydroelectrolytic balance—commonly for the classic diuretic agents (Hooman et al., 2009; Titko et al., 2020). Classical diuretic agents usually act by causing a strong inhibition of several key cotransporters in the nephron, with a significant reduction of electrolyte and water reabsorption which leads to an intense diuretic and saluretic effect but also to sometimes severe adverse effects. Herbal diuretics could also enhance the urinary excretion of electrolytes and water, but their effect has a lower magnitude and is more gradually installed, thus having a reduced risk of causing adverse effects (Wile, 2012).

The results obtained after chemical and biological assessment of TCI, TCT and OpTC highlight the positive impact of the hydroethanolic solvent and microwave-assisted extraction as processing factors which can improve the quality of *T. comosus* herbal preparations. The use of herbal remedies in ethnopharmacology is associated with simple, conventional processing methods, which do not require special conditions, solvents and equipment, mainly suitable for household use (i.e., infusion, maceration, decoction, mechanic processing of raw fresh material). Recognized for their accessibility, these methods have some limitations regarding the proper recovery of bioactive compounds from the plant material and prolonged extraction times. As it could be observed, TCI and OpTC showed a similar diuretic and antimicrobial potency, which suggest that the extraction of main bioactive compounds from *T. comosus* can be improved using microwave-assisted extraction by decreasing extraction times and solvent consumption; this statement is also supported by the quantitative distribution of main phenolic classes in the aforementioned extracts (see Table 1), all of them (excepting phenolic acids and flovan-3-ols) reaching the highest amounts in OpTC. Moreover, this findings highlight the ability of our previous–established ultrasound-assisted extraction method (Babotă et al., 2022) to increase the recovery of phenolic compounds from *T. comosus* aerial parts in comparison with classic extractive methods (i.e. infusion, maceration).

## 5 Conclusion

Our present work emphasizes for the first time the *in vivo* diuretic potential of phenolic-rich herbal preparations obtained from the aerial parts of *T. comosus*, a wild thyme species endemic for Romanian spontaneous flora. The good results obtained after the *in vitro* evaluation of antimicrobial activity support the potential use of this species as adjuvant therapy for urinary tract infections due to its dual action—mild diuretic and antibacterial, previously suggested by the ethnopharmacological evidence. Moreover, these proven bioactivities were correlated with the occurence of several phenolic classes confirmed after UHPLC-QTOF-HRMS screening of the analyzed extracts. Additionally, the influence of different extraction procedures against the phytochemical and bioactive profile of herbal preparations obtained from *T. comosus* was evaluated, proving the ability of ultrasound-assisted extraction to improve the recovery of main phenolic constituents of this species in comparison with classic extractive methods. All data provided by our study encourage supplementary evaluations for OpTC extract in order to unravell the deepen pharmacological mechanisms responsible for its bioactivity, which can constitute a solid basis for the potential future development of dietary supplements or phytomedicines using this extract.

## Data Availability

The original contributions presented in the study are included in the article/Supplementary Material, further inquiries can be directed to the corresponding author.
